# Supplementary figures and images for: “Sports for All”—An Evaluation of a Community Based Physical Activity Program on the Access to Mainstream Sport for Children with Intellectual Disability
Source: Int J Environ Res Public Health. 2022 Sep 14;19(18):11540. doi: 10.3390/ijerph191811540 (PMC9517241; doi:10.3390/ijerph191811540)

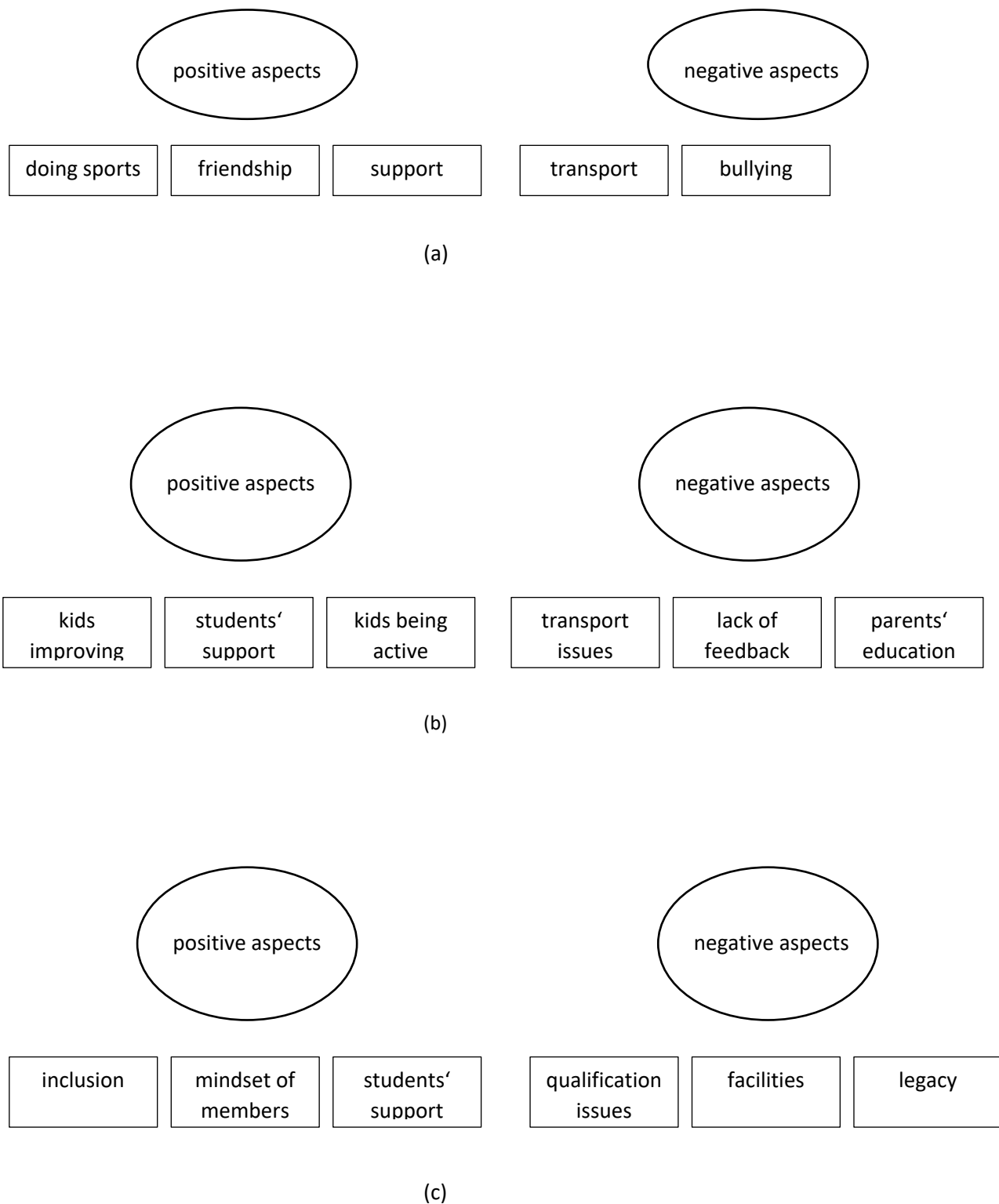

**Figure S1.** Final thematic maps showing the main themes of (a) kids; (b) parents; (c) clubs.

Supplement: Supplementary file 1 [file ijerph-19-11540-s001.zip › ijerph-1869102-supplementary.pdf]
